# Risk Factors and Prognosis of Peritoneal Metastases in Small Bowel Neuroendocrine Tumor Patients

**DOI:** 10.1245/s10434-025-18374-7

**Published:** 2025-09-25

**Authors:** Jeremy Chang, Luis C. Borbon, Scott K. Sherman, Po Hien Ear, Andrew M. Bellizzi, Joseph S. Dillon, James R. Howe

**Affiliations:** 1 Department of Surgery, University of Iowa Carver College of Medicine, Iowa City, IA USA; 2 Department of Pathology, University of Iowa Carver College of Medicine, Iowa City, IA USA; 3 Department of Internal Medicine, University of Iowa Carver College of Medicine, Iowa City, IA USA

## Abstract

**Background:**

Peritoneal metastases (PM) are common in small bowel neuroendocrine tumors (SBNETs). Risk factors for PM and their impact on disease prognosis is poorly studied.

**Objectives:**

This study reviews 20 years of experience with SBNETs, focusing on identifying risk factors for PM and the long-term oncologic outcomes of cytoreduction.

**Methods:**

A retrospective single institutional study was performed from 2005 to 2024 identifying patients with SBNETs who underwent operative resection of their tumors. Primary endpoints were overall survival and progression-free survival, assessed with the Kaplan–Meier method.

**Results:**

In total, 396 patients were included in the study with rates of metastatic disease and PM of 71.7 and 30.1%, respectively. The presence of liver metastases, female sex, higher T stage, and elevated pancreastatin levels were associated with an increased risk of PM. By Kaplan–Meier analysis, patients with PM had worse overall survival (109 vs. 169 months; *p *= 0.01) and progression-free survival (42 vs. 75 months, *p *<0 .01) than those without PM; however, on multivariable analysis, these were not significant. Patients who received cytoreduction of PM had higher rates of major complications (i.e. Clavien–Dindo 3 or 4) than those who did not. Additionally, patients with PM had higher rates of postoperative bowel obstruction, although the greater use of peptide receptor radionuclide therapy in this population may also have contributed.

**Conclusions:**

Peritoneal metastases are common in SBNET. Aggressive surgical management is associated with favorable long-term survival, which suggests it should be considered.

Small bowel neuroendocrine tumors (SBNET) are the most common primary tumor of the small intestine.^1^ Although typically classified as indolent and slow growing, SBNETs have a high propensity for metastasis and extraregional spread, most commonly to the liver. The peritoneum is the next most common site, which may arise from tumors growing through the serosa of the small bowel and shedding metastatic cells throughout the peritoneal cavity. The incidence of peritoneal metastases (PM) has been reported as 10–30% in larger series,^2–6^ and they may lead to reduced overall survival (OS).^6^ PM can produce many undesirable outcomes for patients. They may implant on the bowel wall, causing narrowing or kinking of the bowel, leading to abdominal pain and small bowel obstructions. They often occur in dependent areas such as the diaphragm or pelvis, the latter occasionally leading to rectosigmoid obstruction. On exploration, there can be hundreds of small lesions scattered about the mesentery and peritoneum, sometimes causing ascites, and larger lesions can narrow blood vessels or cause ureteral obstruction.

Preoperative diagnosis of PM can be challenging. Larger lesions (>1 cm) can be identified on computed tomography (CT) scan, but subcentimeter lesions may be missed. The addition of somatostatin receptor DOTA-positron emission tomography (PET)/CT imaging has improved sensitivity for detecting PM, but they may still elude discovery, and false-positive findings also occur.^7,8^ Diagnosing the presence of PM preoperatively helps with operative planning, as attempting to remove these sites of disease requires more extensive surgery.

Oncologic resection is a mainstay in the management of SBNETs, and the presence of PM does not preclude surgery. The North American Neuroendocrine Tumor Society guidelines recognize that there is no effective treatment for PM other than early resection of primaries to prevent their development and to cytoreduce tumor implants when present. These guidelines recommend “removing as much disease as possible while minimizing risks.”^9^ As patients with SBNETs often have prolonged survival, symptom control must also be factored into disease treatment. Although studies are few, several have shown that cytoreduction of PM, often in combination with hepatic metastases, can improve oncologic survival outcomes.^4,5,10–12^ Herein, we review 20 years of experience with SBNETs, identifying risk factors for PM in patients with SBNET and exploring whether PM is associated with decreased progression-free survival (PFS) and OS.

## Methods

### Study Population

We performed a retrospective review of a prospectively maintained database from a single academic medical center, with patients from 2005 to 2024. Patients were included if they (1) had histopathologic diagnosis of SBNET and (2) underwent operative resection of primary tumor ± metastases (where appropriate). Only tumors originating from ileal or jejunal primary sites were included; duodenal neuroendocrine tumors (NETs) were not included as this subgroup may be biologically distinct and behave differently clinically.^13,14^ Patients were excluded if they had more than one primary site of NET (e.g., SBNET and pancreatic NET) or multiple lifetime cancer diagnoses. Patient demographic, pathologic, treatment, postoperative complications, and oncologic outcomes data were abstracted. Extent of peritoneal disease was classified based on the Lyon score according to lesion size and abdominal localization.^15^ Primary study outcome measures were OS and PFS. Both OS and PFS were calculated from the time of first operation. PFS data were obtained through evaluation of follow-up clinic notes and surveillance imaging. OS end date was obtained from patient recorded death date (where applicable) or last known clinic follow-up for censored patients. Characterization of disease progression was based on Response Evaluation Criteria in Solid Tumors (RECIST) criteria.^16^ Secondary outcome measures were postoperative complication rates, based on the Clavien–Dindo classification.^17^ Incidence of postoperative bowel obstruction and timing of receipt of peptide receptor radionuclide therapy (PRRT) were also studied. This study was performed in compliance with the guidelines of the University of Iowa Human Subjects Office under an approved institutional review board protocol (IRB#199911057).

### Statistical Analysis

Summary statistics were used to describe the study population. Categorical variables were compared with the chi-squared test, and continuous variables were compared with the Mann–Whitney U test. The Kaplan–Meier method provided estimates of OS and PFS. Kaplan–Meier survival curves were compared for significance with the log-rank test. Right censoring was used for survival analysis for patients alive at the end of the study period. The minimum follow-up opportunity for patients was 1 year. Univariate and multivariable Cox hazard ratio analyses were utilized to identify variables associated with positive or negative effects on OS or PFS. Univariable and multivariable regressions were used to investigate the association of variables with PM occurrence and the complication rate. Demographic, cancer, and treatment variables were included in the multivariable analysis. Analyses were performed with R via R Studio.

## Results

### Study Population

A total of 396 patients with SBNET were included in the study (Table 1). The median follow-up was 81 months (95% confidence interval [CI] 73–91). Seven patients with concurrent diagnoses of gastric adenocarcinoma, ampullary adenocarcinoma, cholangiocarcinoma, ovarian carcinoma, or leiomyosarcoma were excluded. All patients received cytoreductive surgery at our institution. In total, 284 (71.7%) patients had metastatic disease on initial presentation, 266 (67.1%) with liver metastases and 18 (4.0%) with isolated PM. Twelve patients did not receive cytoreduction of liver metastases because of diffuse liver involvement. A total of 119 (30.1%) patients had PM over their disease course. Of these, 21 (5.3%) did not have PM at the time of their first NET surgery but developed PM on progression. The operative strategy in patients with PM was to reduce the volume of disease as much as possible. Partial omentectomy was performed for lesions in this location. For lesions on the bowel wall, these were frequently shaved off and the serosa reinforced with sutures. For mesenteric implants, those larger than 5–10 mm were commonly removed, whereas those <5 mm were treated with argon beam coagulation. For lesions in the pelvis or other peritoneal surfaces, the same general strategy was used with respect to size; larger lesions were removed with the underlying peritoneum (which was not formally stripped) and smaller lesions treated with the argon beam coagulator. If the rectosigmoid was significantly narrowed by tumor, then low anterior resection was performed. If ureters were involved, they were resected and primarily re-anastomosed. When ovaries were involved, oophorectomy was performed. Smaller (< 5–10 mm) diaphragmatic lesions were treated with the argon beam coagulator, and larger lesions were excised, with large lesions or caking requiring excision and primary repair and a pigtail chest tube.

**Table 1:** Comparison of characteristics between patients with and without peritoneal metastases (PM)

Characteristic	PM (n = 119)	No PM (n=277)	*p*-Value
Median age	59 (52–67)	59 (52–68)	0.6
Sex			<0.01
Male	49 (41.2)	163 (58.8)	
Female	70 (58.8)	114 (41.2)	
ASA class			0.02
1	2 (1.7)	3 (1.1)	
2	48 (40.3)	111 (40.0)	
3	42 (34.5)	132 (47.7)	
4	6 (5.0)	3 (1.1)	
Missing	21	28	
Synchronous liver metastasis	105 (88.2)	189 (68.2)	<0.01
Grade			0.21
1	47 (39.5)	133 (48.0)	
2	63 (52.9)	127 (45.8)	
3	7 (5.9)	10 (3.6)	
Missing	2	7	
Primary tumor size			<0.01
<1 cm	13 (10.9)	27 (9.7)	
1–2 cm	31 (26.1)	136 (49.1)	
>2 cm	64 (53.8)	97 (35.0)	
Missing	11	17	
T stage			<0.01
0	0	2 (0.7)	
1	3 (2.5)	9 (3.2)	
2	6 (5.0)	52 (18.8)	
3	41 (34.5)	133 (48.0)	
4	54 (45.4)	64 (23.1)	
Missing	15	17	
N stage			0.18
0	4 (3.4)	17 (6.1)	
1	72 (60.5)	181 (65.3)	
2	38 (31.9)	67 (24.2)	
Missing	5	12	
Neuroendocrine markers			
Preop serotonin	1201 (757–1753)	824 (333.5–1320)	<0.01
Preop chromogranin A	221.5 (99–642)	210.5 (91.5–615.5)	0.6
Preop neurokinin	17 (10–29)	16 (8–28)	0.54
Preop pancreastatin	551 (251.5–1023.5)	291 (101–902)	<0.01
Additional treatments			
PRRT	43 (36.1)	55 (19.9)	<0.01
SSA	97 (81.5)	189 (68.2)	0.01
Chemo/biologic	12 (10.1)	29 (10.5)	0.91

Data are presented as median (interquartile range) or n (%) unless otherwise indicated

ASA, American Society of Anesthesiologists; preop, Preoperative; PRRT, Peptide receptor radionuclide therapy; SSA, Somatostatin analog

Patients with PM were more frequently female and had higher rates of synchronous liver metastases, larger primary tumor size, higher T stage, and higher levels of preoperative serotonin and pancreastatin than patients without PM (Table 1). There was no significant difference in tumor grade or N stage between those with and without PM. Interestingly, patients with no PM were categorized as at a higher American Society of Anesthesiologists class than those with PM. Most patients with PM had diffuse disease, and 79/94 (84.0%) cases had a Lyon score of 3 or 4 (25 patients were excluded because information was missing). The median follow-up was longer for patients with PM than for those without (87 vs. 77 months, respectively; *p *< 0.01). Regarding subsequent treatments, patients with PM more frequently had PRRT (36.1 vs. 19.9%; *p *< 0.01) and somatostatin analogs (81.5 vs. 68.2%; *p *= 0.01) than those without PM. There was no difference in the frequency of use of chemotherapy/biologic agents (~10% in both groups).

### Risk Factors for PM

Univariable and multivariable regression were performed to identify risk factors for PM development. On univariate analysis, female sex, presence of liver metastases, T stage 3 or 4, preoperative elevation of serotonin and/or pancreastatin were risk factors for developing PM (Table 2). On multivariable analysis, the presence of synchronous liver metastases, female sex, T stage 3 or 4, and elevated preoperative pancreastatin were independent risk factors for developing PM. In total, 21 patients developed PM later in their disease course: 18 had PM identified on surveillance imaging, and three had PM identified on subsequent surgery (one for cytoreduction, one for a hernia repair, and one for bowel perforation). The median time to PM diagnosis in this group was 54 months post-surgery (95% CI 39–105).

**Table 2:** Univariable and multivariable odds ratio analysis for risk factors for development of peritoneal metastases (PM)

Risk factor	Univariate regression		Multivariable regression	
	OR (95% CI)	*p*-Value	OR (95% CI)	*p*-Value
Age >65 years (ref: age ≤65)	0.96 (0.87–1.07)	0.48		
Male sex (ref: female sex)	0.86 (0.79–0.94)	<0.01	0.82 (0.75–0.91)	<0.01
Synchronous liver metastasis	1.26 (1.13–1.39)	<0.01	1.16 (1.03–1.29)	0.01
Grade (ref: 1)				
2	1.07 (0.98–1.18)	0.13		
3	1.16 (0.92–1.45)	0.22		
Primary size (ref: <1 cm)				
1–2 cm	0.87 (0.75–1.02	0.09		
>2 cm	1.08 (0.92–1.26)	0.36		
T3 or T4 (ref: T1/T2)	1.22 (1.08–1.37)	<0.01	1.15 (1.02–1.29)	0.02
N stage (ref: N0)				
N1	1.11 (0.90–1.36)	0.36		
N2	1.19 (0.96–1.48)	0.11		
Elevated pre-op neuroendocrine labs				
Serotonin (nl <200 ng/ml)	1.19 (1.05–1.36)	0.01	1.09 (0.95–1.25)	0.25
Chromogranin A (nl <187 ng/ml)	1.01 (0.92–1.11)	0.853		
Neurokinin A (nl <40 mg/ml)	0.99 (0.86–1.15)	0.93		
Pancreastatin (nl <136 pg/ml)	1.28 (1.15–1.42)	<0.01	1.16 (1.04–1.31)	0.01

CI, Confidence interval; OR, Odds ratio

### Impact of PM on Oncologic Outcomes

**Fig. 1:**
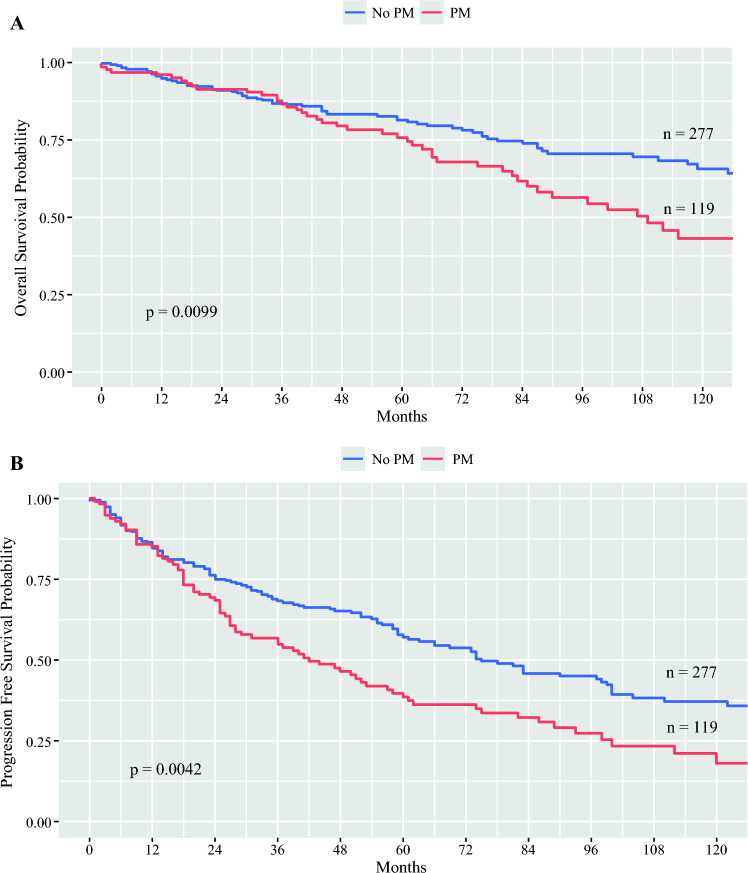
Kaplan–Meier survival curves for **A** overall survival and **B** progression-free survival comparing patients with and without peritoneal metastases (PM)

**Fig. 2:**
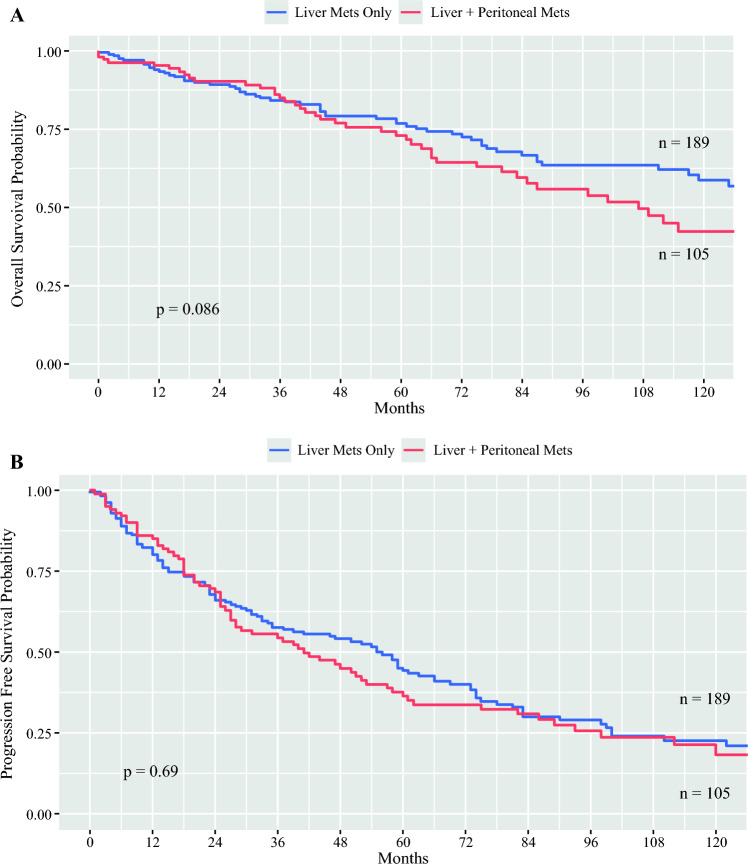
Kaplan–Meier survival curves for **A** overall survival and **B** progression-free survival comparing patients with liver metastases with or without peritoneal metastases (PM). Mets, metastases

The median OS for the entire study population was 145 months (95% CI 128–203), and the median PFS was 69 months (95% CI 58–83). Kaplan–Meier survival curves for OS and PFS were generated for patients with PM versus no PM (Fig. 1). Patients with PM had worse OS (109 vs. 169 months; *p *= 0.01) and PFS (42 vs. 75 months; *p *< 0.01) than those without PM. Univariable and multivariable Cox hazard ratio analyses were performed to identify independent variables affecting OS and PFS. Age > 65 years, presence of liver metastases, PM, and grade 2 or 3 disease (relative to grade 1 disease) were associated with worse OS on univariable analysis; age, liver metastases, and grade remained significant on multivariable analysis (Table 3). The presence of liver metastases, PM, and grade 2 or 3 disease was associated with worse PFS on univariable analysis; liver metastases and grade remained significant on multivariable analysis (Table 4). Although the presence of PM was associated with worse OS and PFS on univariate analysis, this finding was not a significant independent risk factor on multivariable analysis. To explore this further, we compared patients with liver metastases and PM and those with liver metastases but without PM. The median OS in these two groups was 107 months (95% CI 83–145) and 146 months (95% CI 125–not reached), respectively (*p *= 0.09). The median PFS was 41 months (95% CI 28–58) and 56 months (95% CI 37–66), respectively (*p *= 0.69; Fig. 2). As suggested by the earlier analysis, PM was not a significant independent factor for OS or PFS in patients with liver metastases.

**Table 3:** Univariable and multivariable Cox hazard ratio analysis of risk factors for overall survival

Risk factor	Univariable		Multivariable	
	HR (95% CI)	*p*-Value	HR (95% CI)	*p*-Value
Age >65 years (ref: age ≤65)	1.99 (1.37–2.90)	<0.01	1.96 (1.34–2.88)	<0.01
Male sex (ref: female sex)	0.92 (0.65–1.32)	0.66		
Liver metastases	2.98 (1.60–5.54)	<0.01	2.58 (1.36–4.87)	<0.01
Peritoneal metastases	1.50 (1.04–2.16)	0.03	1.24 (0.85–1.81)	0.26
Lyon score (ref: 1)				
2	0.78 (0.19–3.20)	0.73		
3	0.52 (0.15–1.78)	0.29		
4	0.44 (0.12–1.58)	0.21		
Grade (ref: 1)				
2	1.67 (1.14–2.46)	0.01	1.61 (1.09–2.37)	0.02
3	3.68 (1.79–7.57)	<0.01	2.79 (1.34–5.82)	0.01
T3 or T4 (ref: T1/T2)	1.25 (0.76–2.03)	0.38		
N stage (ref: N0)				
N1	0.82 (0.41–1.64)	0.58		
N2	1.24 (0.57–2.71)	0.59		
Postoperative complication	1.08 (0.69–1.70)	0.73		

CI, Confidence interval; HR, Hazard ratio

**Table 4:** Univariable and multivariable Cox hazard ratio analysis of risk factors for progression-free survival

Risk factor	Univariable		Multivariable	
	HR (95% CI)	*p*-Value	HR (95% CI)	*p*-Value
Male sex (ref: female sex)	1.18 (0.89–1.56)	0.26		
Liver metastases	3.92 (2.44–6.30)	<0.01	3.80 (2.34–6.15)	<0.01
Peritoneal metastases	1.47 (1.10–1.96)	0.01	1.15 (0.86–1.54)	0.34
Lyon score (ref: 1)				
2	0.76 (0.20–2.91)	0.69		
3	0.56 (0.17–1.85)	0.34		
4	0.62 (0.19–2.10)	0.45		
Grade (ref: 1)				
2	1.57 (1.17–2.10)	<0.01	1.46 (1.10–1.95)	0.01
3	2.08 (1.00–4.32)	0.05	1.75 (0.90–3.42)	0.1
T3 or T4 (ref: T1/T2)	1.22 (0.84–1.78)	0.29		
N stage (ref: N0)				
N1	1.01 (0.54–1.86)	0.99		
N2	1.77 (0.92–3.40)	0.09		

CI, Confidence interval; HR, Hazard ratio

### PM and Disease Morbidity

All patients in this study underwent cytoreduction of their disease (i.e. primary ± metastases), with the exception of 12 patients with widespread liver metastases where it was deemed that ≥70% cytoreduction would not be possible. At the initial surgery, PM were present in 98 (24.7%) patients. Patients with PM had higher rates of postoperative complications than those without PM (25.5 vs. 15.8%; *p *= 0.03) but an overall lower American Society of Anesthesiologists class. These patients had higher rates of major (i.e. Clavien–Dindo 3 or 4) complications (11.2 vs. 2.0%; *p *< 0.01; Table 5). Of note, there were also three cases of postoperative in-hospital mortality (two in the PM group and one in the no PM group). The specific causes of death were a postoperative day 2 cardiac arrest, an anastomotic leak leading to a prolonged course with withdrawal of care, and renal failure leading to multisystem organ failure with withdrawal of care. The most frequent major complications for the entire population included 11 (2.8%) intraabdominal infections requiring drainage (operative or interventional radiology), two (0.5%) ureteral injuries, one (0.3%) anastomotic leak, and one (0.3%) intraoperative cardiac arrest (with rescue). Looking specifically at surgical site infections (SSIs), there was no difference in the rate of superficial SSI; however, patients with PM who underwent cytoreduction of their disease had significantly increased rates of organ space SSI (6.1 vs 1.7%; *p *= 0.02).

**Table 5:** Comparison of postoperative complications between patients with and without peritoneal metastases (PM)

Complications	Surgery with PM (n=98)	Surgery without PM (n=298)	***p*-Value**
Any complication	25 (25.5)	47 (15.8)	0.03
Clavien–Dindo 1/2	14 (14.3)	40 (13.4)	0.92
Clavien–Dindo 3/4	11 (11.2)	6 (2.0)	<0.01
Clavien–Dindo 5	2 (2.0)	1 (0.3)	0.09
Superficial SSI	4 (4.1)	8 (2.7)	0.48
Intraabdominal SSI	6 (6.1)	5 (1.7)	0.02

Data are presented as n (%) unless otherwise indicated

SSI, Surgical site infection

Patients with PM had higher rates of postoperative bowel obstruction (defined as bowel obstruction occurring at any period after surgery) than those without PM (22.7 vs 3.6%; p < 0.01). For patients with PM, we sought to investigate whether there was any association with receiving PRRT, as patients with PM more frequently had PRRT. Patients with PM who were treated with PRRT had significantly higher rates of bowel obstruction than those who were not treated with PRRT (44.2 vs. 10.5%; *p *< 0.01). Of patients who were treated with PRRT and had a postoperative bowel obstruction, 76.2% had an episode of bowel obstruction after receiving both surgery and PRRT, whereas 23.8% of patients had bowel obstructions after surgery but before PRRT. This latter group was not included in the percent of PRRT-treated patients who had a potentially PRRT-associated bowel obstruction as these were before the PRRT. Additionally, among the 298 patients who did not have PRRT, patients with PM had higher rates of bowel obstruction than those without PM (10.5 vs. 4.1%; p = 0.04). Of the 98 patients who received PRRT, those who had PM had higher rates of bowel obstruction than those without PM (44.2 vs. 3.6; *p *< 0.01). Of 43 patients with PM who underwent PRRT, three had bowel obstruction within 3 months of their last PRRT dose. Many of these patients had multiple episodes of bowel obstruction, with a median time to the first episode of bowel obstruction after receiving PRRT of 17 months (95% CI 15–61). Patients with PM who were treated with PRRT required more frequent operative intervention to relieve bowel obstructions than patients with PM not treated with PRRT (34.9 vs. 3.9%; *p *< 0.01). Of the 15 patients with PM who received PRRT and then required surgical intervention, six required bowel resections, five had lysis of adhesions only, three had diverting ostomies, one had palliative gastrostomy, and one had a colonic stent.

## Discussion

Few studies describe outcomes of patients with SBNET with PM. Given the longevity of most of these patients, it is important to characterize morbidity as well as oncologic outcomes. This study provides long-term data on a large cohort of patients with SBNET with PM. The rate of PM in this study was 30.1%. The PM incidence rate in the current study is higher than that in national cancer registries. Data from the Netherlands Cancer Registry and from a multicenter Spanish study have described the incidence of PM in their SBNET populations as around 13%.^18,19^ However the incidence rate is similar to that in other tertiary referral centers.^4,5^

Risk factors for developing PM remain poorly studied. In our multivariable regression, the presence of liver metastases, higher pancreastatin, higher T-stage, and female sex were associated with an increased risk of PM. Das et al.^20^ investigated risk factors for PM development in 208 patients and found that higher T stage (T3/T4) and presence of mesenteric tumor deposits were associated with an increased risk of PM development. Although female sex was associated with an increased risk of PM, this does not necessarily portend worse OS, as many recent studies have found that females diagnosed with SBNETs tend to have longer survival than males.^21,22^ NET biomarkers have been recognized as correlated with disease burden and may be predictive of progression.^23–25^ Chromogranin A is the most widely used and the only marker recommended in the National Comprehensive Cancer Network guidelines,^26^ but elevated pancreastatin is associated with worse PFS and OS in patients with SBNET.^27^

In our study, we found that the median time to PM progression was 54 months, which supports a long duration of follow-up. Current National Comprehensive Cancer Network guidelines for surveillance recommend CT or magnetic resonance imaging every 1–2 years for patients with locoregional disease and CT or magnetic resonance imaging every 3 months to 1 year for those with metastatic disease, both for at least 10 years.^28^ For the majority of patients, even those with metastases, surveillance imaging every 3 months may not be necessary except in those with higher-grade tumors. CT scans have relatively good sensitivity (80–85%) and specificity (82–86%) for identifying larger peritoneal deposits.^29,30^ However, with lesions < 1 cm in size, sensitivity decreases to 7–28%. Somatostatin receptor DOTA-PET/CT may be helpful in disease follow-up, but its use is not routinely recommended and should be at the discretion of the provider. ^31^ We find this modality to be particularly helpful in the preoperative evaluation of all patients who are to undergo surgery, as it commonly identifies additional sites of metastasis, including PM, not seen by other methods, which helps operative planning.

The effect of PM on the survival of patients with SBNET is not agreed upon in the literature. Madani et al.^19^ evaluated 4114 patients in the Netherlands Cancer Registry and found that the presence of PM in the subset of patients with metastatic disease was not associated with worse survival. These findings differed from those of Wright et al.^32^ who, in their analysis of 219 patients with SBNET, found that those with both liver metastases and PM had worse survival than patients with only liver metastases. However, their study did not report the proportion of patients that had cytoreduction of their metastatic disease. Norlen et al.^33^ found that 17% of their patients with SBNET had PM, and 5-year survival was 79% in those without PM and 52% in those with PM. PM was an independent risk factor for reduced OS, but it was unclear how the PM were treated, and only 35% of patients with liver metastases had debulking procedures.^33^ In studies where cytoreduction of peritoneal disease was performed, there was no difference in long-term survival of patients with liver metastases and PM compared with that of patients with liver metastases alone.^4,34^ In the present series, although PM was associated with worse OS and PFS in Kaplan–Meier analysis, this relationship was not independently significant on multivariable analysis. When we looked at patients with liver metastases with and without PM, the presence of PM was not associated with worse OS or PFS, further supporting the likelihood that liver metastasis, and not PM, is the major determinant of survival. This finding agrees with prior studies analyzing peritoneal cytoreduction. In a similar population treated with cytoreduction, Wonn et al.^4^ found a median OS of 67 months in 98 patients with PM (54% presenting with liver metastases). They also showed that Lyon stage on closing was predictive of survival.^4^ The median OS in our population with PM (88% with liver metastases) was favorable at 109 months after cytoreduction. Taken together, these data reinforce that aggressive surgical management of patients with SBNET with PM can lead to long-term survival, albeit still worse than those without metastatic disease.^4,5,32^

Although aggressive management of PM can improve oncologic outcomes, this comes with increased perioperative morbidity. Patients undergoing cytoreduction of PM have increased rates of major complications, especially organ space SSI, likely secondary to an increased extent of surgery. Our rate of 2% mortality and 11% Clavien–Dindo class 3+4 complications is similar to the findings of Wonn et al.^4^ of 3 and 15%, respectively. Additionally, patients with PM have higher utilization of adjuvant treatments, including somatostatin analogs and PRRT, than those who do not have PM. Long-term morbidities included increased rates of bowel obstruction in patients with PM, as well as increased likelihood of surgical intervention in patients with bowel obstruction who received PRRT. The extent to which these complications result from the interventions performed or reflect higher disease burden remains for further investigation.

PRRT has been suggested to be associated with bowel obstruction by inducing or exacerbating mesenteric or peritoneal inflammation.^35,36^ In the NETTER-1 trial, no cases of bowel obstruction were reported within the initial follow-up period. However, shortly after the trial, Strosberg et al.^35^ reported that, of 81 patients with mesenteric or peritoneal disease treated with Lutathera, five had one or more episodes of bowel obstruction within 3 months of therapy and recommended treatment with corticosteroids for prophylaxis to decrease inflammation. Merola et al.^37^ found that, of 32 (21.9%) patients with gastroenteropancreatic NETs and PM treated with PRRT, seven developed bowel obstructions, and 37.5% experienced disease progression after PRRT.^37^ Our data support this increased risk of bowel obstruction. Although 6.9% of patients with PM receiving PRRT had a bowel obstruction within 3 months of PRRT, 44.2% of patients with PM who underwent PRRT had at least one episode of bowel obstruction afterwards, with 25.6% of them occurring at least 1 year after PRRT. Not surprisingly, receiving PRRT was also associated with an increased frequency of operative intervention for obstruction.

Studies investigating the addition of hyperthermic intraperitoneal chemotherapy (HIPEC) are limited. Only two retrospective studies have compared cytoreduction alone and cytoreduction plus HIPEC in patients with SBNET with PM. The HIPEC group experienced no survival benefit but significantly higher complication rates,^38,39^ so HIPEC is not currently recommended for patients with SBNET with PM.^40^

Our study is limited by sample size and its retrospective nature. Additionally, all the included patients received surgery, so we were unable to compare outcomes between the surgery group and non-surgical patients. Furthermore, we did not prospectively record the completeness of PM resection or degree of hepatic cytoreduction achieved in these patients. This was a single surgeon experience, and the same operative strategy for removing PMs was used in all patients, with an aim of ≥70% hepatic cytoreduction where possible. Also, not all patients who progressed and developed metastatic disease (either liver or peritoneal) were reoperated upon. These data were obtained from a single academic institution, and the incidence of metastatic disease and rate of adjuvant treatment utilization was higher than in most large databases.

The peritoneum is a common site of extraintestinal disease spread in SBNET. In 2025, the Peritoneal Surface Malignancy Consortium Group published updated guidelines for the management of PM in patients with NET. They emphasized that an understanding of tumor biology and operative and patient goals are critical in caring for these patients.^41^ Operative cytoreduction is recommended in patients with G1 or G2 NETs with surgically treatable disease burden. The presence of PM should not preclude patients from operative management, and appropriate aggressive cytoreduction may improve long-term oncologic outcomes.^34^ Wonn et al.^4^ showed that reducing the Lyon score to <3 led to improved survival, and we believe in removing larger nodules wherever possible and treating smaller lesions with ablation (using argon beam cautery or other methods). The potential for increased morbidity must always be considered when removing individual lesions, performing additional small bowel or rectosigmoid resection, ureteral resection and reanastomosis, and diaphragmatic resection and repair. Management of these patients can be a complex multidisciplinary effort. We have found that preoperative functional somatostatin receptor imaging to understand disease location and burden can be instrumental in guiding operative decision making. Since medical management is generally ineffective in reducing tumor volume, we advise that surgical cytoreduction be employed when operating on patients with SBNETs and PM.^37^
